# Special Issue “Advanced Engineering Cementitious Composites and Concrete Sustainability”

**DOI:** 10.3390/ma16072582

**Published:** 2023-03-24

**Authors:** Dumitru Doru Burduhos-Nergis

**Affiliations:** Faculty of Materials Science and Engineering, Gheorghe Asachi Technical University of Iasi, 700050 Iasi, Romania; doru.burduhos@tuiasi.ro

Concrete, one of the most often-used building materials today, is the cornerstone of modern buildings all over the world, being used for foundations, pavements, building walls, architectural structures, highways, bridges, overpasses, and so on. Because of its adaptability, concrete may be found in practically every construction, in some form or another. Yet, the diverse nature of its components, their combinations, and their doses result in a very wide range of concrete kinds with varying properties. As a result, concrete is a material that is always evolving and is popular even now, especially when it comes to circular economy.

Other ways of concrete manufacturing are now being researched to lessen or remove the limits of this material, which are connected to its brittleness and poor environmental effects. As a result, the development of engineering cementitious composites has resulted in a significant reduction in flexibility issues, while the introduction of new additives and the optimization of the manufacturing process has resulted in a significant reduction in the negative effects of virgin raw material exploitation. In-depth research is still required to optimize and increase the sustainability of these advanced engineering cementitious composites or alternative concretes.

In this Special Issue (SI), state-of-the-art research and review articles on the emerging material systems for AM are collected, with a focus on the process–structure–properties relationships. In total, eleven research papers and six reviews have been collected. Considering the high interest in this field for finding alternatives for virgin raw materials, in the research article conducted by Lv, Z. et al. [1], an interesting experimental study was conducted on the effect of replacing ordinary sand with desert sand on the obtainment and characterization of engineered cementitious materials. Additionally, A. İ. Çelik et al. [2] observed that a 20% replacement of fine aggregates and coarse aggregates with recycled crushed glass resulted in a significant increase in the mechanical properties of concrete. In another study, Ö. Zeybek et al. [3] evaluated the effect of replacing cement with fine glass microparticles on the tensile and flexural strengths of concrete, and showed that a 10% replacement would result in better mechanical properties. Burduhos Nergis, D.D. et al. [4] evaluated the possibility of obtaining acid-activated geopolymers, using mine tailings as a substitute for fine aggregates. In their article, M.I.I. Ramli et al. [5] aimed to obtain alkali-activated ceramics and determined the influence of high curing temperatures on the morphology of kaolin-based geopolymers. To improve the main characteristics of these cementitious composites, some researchers designed and obtained engineered materials by integrating different types of reinforcing elements, or by involving advanced techniques to characterize them. M.H. Yazid et al. [6] obtained geopolymer concrete with improved mechanical performances and water absorption by introducing low amounts of diamond-shaped nylon66 fibers. M.A. Salih et al. [7] incorporated high amounts of supplementary cementitious materials, such as fly ash, ground-granulated blast furnace slag, and microsilica, into self-consolidating concrete, in order to improve the durability and properties of fresh and cured Ordinary Portland Cement (OPC)-based concrete. Z. Yahya et al. [8] developed another self-consolidating concrete for underwater structures and showed that a class C fly ash, activated with a mixture of sodium silicate and sodium hydroxide, could achieve more than 70 MPa when cured in seawater, river water, or lake water. K. Khan et al. [9] showed that ecofriendly concrete could be obtained by replacing OPC with wheat straw ash and/or silica fume. According to their study, this differently engineered composition could achieve better mechanical performances at lower CO_2_-eq. In another study, K. Khan et al. [10] showed that biochar could be used to obtain advanced concrete by performing a SWOT analysis and a techno-economic assessment on the introduction of this by-product as substitute for OPC. Because concrete durability is difficult to assess, particularly for in situ applications, B. Bolborea et al. [11] conducted an experimental investigation on the forecasting of the mechanical properties of concrete, using a non-destructive approach, namely ultrasonic pulse velocity.

In the review articles published in this SI, A.T.M. Yin et al. [12] discussed the potential of producing mold inserts for rapid tooling, using geopolymer composites that were reinforced with recycled metal particles, while N.A.M. Mortar [13] conducted a comprehensive literature analysis on the obtainment and characterization of kaolin-based geopolymers for ceramic applications. R. Martínez-García et al. [14] reviewed the recent developments of the effect produced by the addition of waste wood ash on the composition of different types of concrete. D.L.C. Hao et al. [15] assessed the previous studies on the characterization of artificial aggregates that were manufactured by sintering, cold bonding, or autoclaving, and concluded that the last two methods were suitable for producing lightweight aggregates for industrial use. M. Kheimi et al. [16] presented an overview of the research that was conducted on the parameters that influence the performances of geopolymers that are used in heavy-duty applications, and observed that the mixing design, curing conditions, alkali activator, and binder type are the key factors that define the properties of the final product. A scientometric analysis, considering the publications that are indexed in the Scopus database, was conducted by M.N. Amin et al. [17], in order to establish the statical overview and mapping of the research on rice husk ash utilization in concrete compositions. According to their study, despite the high number of papers published in this field, the lack of standardization in the preparation, process, and use of geopolymers, is the main limitation toward the industrial use of this material.
